# Model Selection from Multiple Model Families in Species Distribution Modeling Using Minimum Message Length

**DOI:** 10.3390/e27010006

**Published:** 2024-12-26

**Authors:** Zihao Wen, David L. Dowe

**Affiliations:** 1 College of Mathematics and Informatics, South China Agricultural University, No. 483, Wushan Road, Tianhe District, Guangzhou 510642, China; 2 Department of Data Science and Artificial Intelligence, Faculty of Information Technology, Monash University, Clayton, VIC 3800, Australia

**Keywords:** minimum message length, model selection, species distribution modeling

## Abstract

Species distribution modeling is fundamental to biodiversity, evolution, conservation science, and the study of invasive species. Given environmental data and species distribution data, model selection techniques are frequently used to help identify relevant features. Existing studies aim to find the relevant features by selecting the best models using different criteria, and they deem the predictors in the best models as the relevant features. However, they mostly consider only a given model family, making them vulnerable to model family misspecification. To address this issue, this paper introduces the Bayesian information-theoretic minimum message length (MML) principle to species distribution model selection. In particular, we provide a framework that allows the message length of models from multiple model families to be calculated and compared, and by doing so, the model selection is both accurate and robust against model family misspecification and data aggregation. To find the relevant features efficiently, we further develop a novel search algorithm that does not require calculating the message length for all possible subsets of features. Experimental results demonstrate that our proposed method outperforms competing methods by selecting the best models on both artificial and real-world datasets. More specifically, there was one test on artificial data that all methods got wrong. On the other 10 tests on artificial data, the MML method got everything correct, but the alternative methods all failed on a variety of tests. Our real-world data pertained to two plant species from Barro Colorado Island, Panama. Compared to the alternative methods, for both the plant species, the MML method selects the simplest model while also having the overall best predictions.

## 1. Introduction

Species distribution modeling (SDM) is an indispensable topic in ecology. It is used to predict and understand the geographic distribution of species based on environmental features. By analyzing data on species occurrences and environmental factors such as climate, habitat, and topography, SDM allows researchers to gain insights into the ecological niche of species and their responses to environmental changes. In the real world, SDM also helps make management decisions such as conservation prioritization and planning [1,2]. Although the argument about the necessity of model selection in SDM exists, in practice it is still one of the most important and commonly used tools due to the nature of ecology study. Tredennick et al. [3] gave an example that we would have to block all seed dispersal on Barro Colorado Island (BCI) while maintaining other processes, and they waited many decades to observe the dispersal effect on canopy tree diversity. It usually requires too many resources and takes too long to conduct manipulative experiments to test a hypothesis of the significance of the features.

In SDM, some of the commonly used model selection techniques include cross-validation [4], regularization [3], significance testing [5], Akaike information criteria (AIC), and Bayesian information criteria (BIC) [6,7,8,9,10]. However, most of these techniques are used for feature selection, given the model family specified by expert knowledge. When reliable expert knowledge is not accessible, misspecification of the model family can be challenging. In this work, we focus on the model selection criteria. Previous studies (e.g., [11]) also point out that AIC tends to select models with an incorrect subset of features if that model has a higher prediction accuracy on training data.

Motivated by a comparison between the AIC and minimum message length (MML) in order selection for polynomial functions [12,13,14], including similar comparisons for autoregression [15], logistic regression [16], moving average models [17], the gappy problem, and more general matters [18], we propose a model selection framework for SDM utilizing MML. Our framework provides robust model selection against model family misspecification, and data aggregation (more specifically, the data are summarized and converted from a fine-grained level of details to a coarser level of details). We also propose a search algorithm for the MML model selection framework that enhances the efficiency of selecting models.

The novelty and contribution of the paper include the following: (i) Adapting MML to two-dimensional spatial point processes; (ii) Using MML for both (ii a) feature selection and (ii b) model selection; (iii) Doing this in a way that is robust to model family misspecification; (iv) We propose a new search algorithm. We have compared with methods that can also do both feature selection and model selection—and we show that MML performed best.

The rest of this article is structured as follows. Section 2 introduces SDM, as well as some of the commonly used model selection techniques in SDM. Section 3 briefly introduces MML. Section 4 presents the MML model selection framework for SDM and the search algorithm. Section 5 conducts simulations for model selection with the correct model family, as well as with model family misspecification. Experiments based on a real-world ecological case are demonstrated in Section 6. And Section 7 concludes the paper.

## 2. Species Distribution Modeling

In species distribution modeling, the survey area is typically divided into grids, with feature data recorded per grid. However, species distribution data can be recorded in different ways. They can be recorded as binary occurrence data that represent the presence and absence of a species in the given grid. Logistic regression is commonly used for this type of data [19]. They can also be recorded as count data per grid. The point processes are fitted to count data instead of Poisson models. This is due to three main problems that interfere with accurate estimation [20] (Chapter 2.3), namely, the boundary problem, the modifiable unit problem, and the pattern problem.

The inhomogeneous Poisson point process (IPPP) is one of the most popular point processes used in SDM [21,22]. An IPPP defined over a survey area *A* has the density function:

(1) $$P\left(N\left(A\right)=n\right)=\frac{\lambda^{n}}{n!}\exp\left(-\lambda \right)$$

where N(A) is the number of occurrences observed in *A*. In an IPPP, the Poisson parameter becomes an intensity function given site *u*. The intensity function could be defined as follows [23]:

(2) $$\begin{array}{c} \lambda \left(u\right)=\lim_{\Delta (u)\to 0}\frac{E(N(u))}{\Delta (u)} \end{array}$$

where Δ(u) is the size of *u*. And the intensity function over the region *A* could be defined as follows:

(3) $$\begin{array}{c} \mu \left(A\right)=\int_{A}\lambda \left(u\right)ds \end{array}$$

and the density function becomes the following:

(4) $$\begin{array}{c} P\left(N\left(A\right)=n\right)=\frac{\mu {\left(A\right)}^{n}}{n!}\exp\left(-\mu \left(A\right)\right) \end{array}$$

Since the IPPP proposes to estimate the intensity function instead of the occurrence probability, it does not need to include the scale.

In many studies, the intensity function would be suggested to have a log-linear (or relaxed using quadratic terms or splines [22]) form, e.g., see [23,24,25].

### Model Selection in Species Distribution Modeling

A previous study [26] concluded that model selection might not be a mandatory step for phylogeny reconstruction. In this study, the authors constructed several experiments to evaluate the utility of various model selection approaches when modeling to predict plant distribution. The scenarios applied in this study are similar to our research on the MML model selection criterion. The authors claimed that applying different model selection approaches results in similar prediction accuracy, and there is no significant improvement when using a specific model selection approach. Moreover, rather than selecting a specific model based on model selection approaches, the authors suggest ignoring this step and choosing only the most complex model and fitting the data, which the authors claim only has a negligible impact on prediction accuracy [26].

Although some details of the experimental design are not clearly mentioned in the published version [26], e.g., whether the explanatory variables used in the experiments have been proven relevant or not to the distribution of the plants, we disagree with the conclusion that choosing the most complex model is the best implementation of model selection. Firstly, choosing the most complex model without enough statistical support is risky: it has a high chance of overfitting. Secondly, we have conducted similar experiments in which several model selection criteria were compared (see Section 5 and Section 6). As a result, the prediction performance outcomes given by different methods are similar, but the inferences vary, i.e., if we generate multiple test sets from the same model and the same set of explanatory variables, the variables selected by AIC were found to vary, while the MML method showed great consistency, and AIC and MML had overall similar prediction performance. In other words, ignoring the model selection criteria and simply selecting the most complex model might lead to no loss of prediction power, but it also might bring risks of losing consistency (i.e., that the model might converge to something incorrect as the amount of data increases), particularly when dealing with multiple datasets.

The target application scenario of this paper is that one can use a model selection technique to select the best subset of features, as well as the best model family, among the given species distribution models. To avoid ambiguity and make the wording consistent, in this paper, the terms ‘best subset’, ‘best model’, and ‘best model family’ refer to the subset, model, or model family that is closest to the one generating the data. If the true model is unknown such that the closeness can therefore not be measured, then the subset/model/model family that is simplest while having prediction accuracy insignificantly different from the best prediction will be called the best. There are many feature/model selection techniques, but only a few are appropriate in the scenario just described.

Some R packages for spatial point processes (e.g., spatstat [27]) and regressions (e.g., glm [28]) use hypothesis testing to measure the significance of features (i.e., modeling with the feature versus modeling without the feature). Such methods perform poorly when collinearity exists among the features, e.g., taking the two individually most important features (assuming that they are highly correlated) and removing them is not necessarily equivalent to removing the most important pair of features. Discussions about MML and hypothesis testing can be found in [29] (Section 1) and [30].

Principal component analysis (PCA) [31] and deep learning autoencoders [32] both do not return the original raw features—but rather combinations (linear for PCA) of them—which is not desired, because we want important raw features rather than combinations of them. Also, they are unsupervised learning methods, and we want features that are important to the target distributions.

Deep feature selection and other neural network-based techniques usually do not allow fitting an inhomogeneous Poisson point process but only an approximate one.

Random forests [33] use an ensemble of decision trees and are popular in both ecology and machine learning. But we are not aware of any approach that uses random forests for the problems under consideration.

MaxEnt [34], a popular model used in ecology, returns the rank of features based on their permutation importance. The selection threshold must be manually chosen, which is not ideal. A discussion comparing MaxEnt with MML is given in [29] (Section 0.2.5, p. 535, col. 1).

As such, information-based criteria (including MML) appear to be the most suitable. Furthermore, compared with AIC, AICc, and BIC, which are the most frequently used information-based criteria in species distribution modeling (SDM), the MML method showed superior performance for simulated SDM data (see Section 5) and real-world SDM data (see Section 6).

## 3. Minimum Message Length

Minimum message length (MML) [35] is a Bayesian information-theoretic principle, which can be thought of in terms of Occam’s razor [36]: there is a quantitative trade-off between the simplicity of the model and the goodness of fit to the data, and (in turn) the model that gives the shortest two-part message is the most probable source of the data. The message consists of two parts: a general assertion about the source of the data and the data itself encoded in a code that would be optimal if the assertion is true [37,38].

MML has a close connection with Kolmogorov complexity, which has been discussed in various places (e.g., [30,37] and [39] (Chapter 2)). This connection of MML with Solomonoff-Kolmogorov complexity means that, broadly speaking and where this is possible, at least in principle and given enough compute time and enough data, MML has a generality and universality which enables it to infer and converge (arbitrarily close) to arbitrarily general computable functions. Kolmogorov complexity is also variously known as Solomonoff–Kolmogorov complexity or algorithmic information theory (AIT), with various relevant writings being [40,41,42,43,44,45,46]. Variants of MML include, e.g., Wallace–Freeman (1987) (or MML87) [37,47], MMLD (or $I_{1D}$) [29] (Section 0.2.2, p. 528, col. 1), [39] (Sections 4.10, 4.12.2, and 8.8.2, p. 360), [48,49,50,51], [52] (Section 6.3, p. 940) and ideal group [29] (Section 0.2.2, p. 529, col. 1), [39] (Sections 4.1, 4.3 and 4.9), [50] (Section 5.2, p. 70, ftn 1), [51] (Section 3.3.3, pp. 60–62), and MML08 [53]. Some examples of the broad generality of MML follow below.

MML has been applied in model selection problems, for example, order selection for polynomial functions [12,13,14], autoregression [15], logistic regression [16], moving average models [17], time series [54], the grouping of ordered data [55,56], and a sequential problem of alternating probabilities ([57], and in [39] (Section 7.3)), etc.

MML has also been used in problems like the inference of decision trees [58], oblique decision trees [59] and decision graphs (allowing ‘OR’ disjunctions to permit joins of branches of decision trees) [39] (Section 7.2) and [60,61,62,63,64], the inference of Bayesian (or causal) networks [39] (Section 7.4) and of Bayesian networks with decision trees in the nodes [65,66], principal component analysis [67], the heterogeneous graphical Granger model [68], type I censoring in exponential distribution [69], the automation of database normalization [70], a shape recognition task [71,72], clustering and mixture modeling [35], [39] (Section 6.8), [73,74,75,76,77], and in the multivariate bounded Kotz mixture model [78], the inference of probabilistic finite-state machines [39] (Section 7.1) and [76], hierarchical probabilistic finite-state machines [79], the analysis of directional data [73,75,80], the analysis of music genres [81], the analysis of protein structure [76,82], etc. MML is also discussed in [83]. Given the abovementioned generality and universality of MML, MML can be applied to inference of neural networks, including (e.g.) Kolmogorov-Arnold networks (KANs).

In most of the model selection studies, MML is used as a model selection criterion given the model family. In practice, using a model selection criterion and comparing all models to select the best one is sometimes intractable, as there might be too many possible models. One Bayesian solution is to use specific priors—such as (e.g.) boosting priors [29] (Section 0.2.6) [52] (Section 6.9) [59] (Section 3.4) and horseshoe priors [84,85]. Horseshoe priors are used for sparse estimation, of which [86] is a case study. Horseshoe priors place weight in the prior to get desired continuous variables. Boosting priors are for multinomial distributions, and put weight on the prior to get more discrete variables. Shrinkage priors are for linear regression. Both boosting and horseshoe priors are to change the model complexity.

Another solution is to combine regularization with the MML method [87]. All of these methods require modification of the target function in estimation and thus need to solve optimization problems. In this paper, we propose a search algorithm that allows using estimators from external sources without modifications.

## 4. MML Model Selection for Species Distribution Modeling

To avoid ambiguity, in this paper, we use the term model selection with regard to selecting models with parameter values from one or more model families. We use the term feature selection with regard to selecting the subset of independent variables, mainly from the given model family.

Suppose that we want to fit a point process with a log-linear intensity function to the species distribution data, which is commonly used in SDM [27] (pp. 299–305). To calculate the message length of the point process, we use the most widely used MML formula, i.e., MML87 (we will sometimes refer to the Wallace–Freeman (1987) MML formula as MML87) [47]. Suppose that the average density of individuals of a species at location *u* is a function λ(u); then, if *u* is small enough, the expected number of individuals at *u* will be λ(u)Δu given that Δu is the area size of *u*. Without considering the interaction between individuals, this point process becomes an IPPP. λ(u) can then be written as follows:

(5) $$\begin{array}{c} \ln\lambda (u)=\theta^{T}S\left(u\right) \end{array}$$

where $\theta =\{\theta_{1},\theta_{2},\ldots ,\theta_{D}\}$ are the coefficients, except $\theta_{1}$ is a constant, and $S\left(u\right)=\{s_{i}\left(u\right)\}$, i=1,…,D are the features at location *u*, except $s_{1}\left(u\right)=1$.

The message length of this IPPP is as follows:

(6) $$\begin{array}{c} I=-\ln\pi \left(\theta \right)+\frac{1}{2}\ln\left|F\left(\theta \right)\right|+\frac{D}{2}\ln\kappa_{D}-\ln f\left(Y|\theta \right)+\frac{D}{2} \end{array}$$

where π(θ) is the Bayesian prior we will discuss in Section 4.1, |F(θ)| is the determinant of the expected Fisher information matrix of θ, f(Y|θ,λ) is the likelihood function, and $Y=(y_{1},y_{2},\ldots ,y_{m})$ are observations. *D* is the number of unknown parameters. $\kappa_{D}$ is the normalized second moment of an optimal quantizing lattice in *D* dimensions [47]. According to the previous study [39] (pp. 257–258), the value of $\kappa_{D}$ can be approximated by the following:

(7) $$\frac{D}{2}\left(\ln\kappa_{D}+1\right)\approx -\frac{D}{2}\ln\left(2\pi \right)+\frac{1}{2}\ln\left(D\pi \right)+\psi \left(1\right)$$

where ψ(1) is a constant equivalent to the Euler–Mascheroni constant, which is approximately 0.5772. In some of the reviewed literature, the optimal quantizing lattice is also called the optimal lattice quantizer. It is the lattice that minimizes the (dimensionless) second moment [88]. The purpose of involving this term in calculating the message length is to find an optimal partition of the parameter space such that, when imprecisely specifying a parameter value, this partition gives the minimum mean squared error. As examples, for D = 2, the optimal lattice is a hexagon; and for D = 3, and the optimal lattice is the body-centered cubic lattice [89]. The lattice constants have known values such as, e.g., $\kappa_{1}=1/12\approx 0.083$, $\kappa_{2}\approx 0.080$, and $\kappa_{3}\approx 0.079$ [90].

### 4.1. The Bayesian Prior Used in MML Calculation

The MML principle is based on the transfer of messages from a sender to a receiver. When the problem involves selecting from multiple model families, the prior probability that the model belongs to a specific family is typically assumed to be equal across families. As a result, the corresponding contribution to the message length from the model family selection can often be neglected. The prior now consists of two parts: the discrete prior represents the feature selection and the continuous prior represents the parameter estimates of the selected features, i.e.,

(8) $$-\ln\pi \left(\theta ,K,D\right)=-\ln h_{s}\left(K,D\right)-\ln h\left(\theta \right)$$

where *K* is the number of candidate features, *D* is the number of selected features, $h_{s}\left(K,D\right)$ is the discrete prior of selecting D out of K features, and h(θ) is the continuous prior of the parameter estimates. The selection of features can be encoded using the following two different methods.

The first method uses K bits (equivalently, K(ln2) nits) to represent the selection, with each bit being a binary number (i.e., 0 or 1) representing the corresponding feature being selected or not. The message length of such a discrete Bayesian prior for this part can be ignored because it is a constant.

The second method is to use $\log_{2}\left(K+1\right)$ bits (equivalently, ln(K+1) nits) that tell us the value of *D* in the range 0,1,2,…,K and then log2KD bits (equivalently, lnKD nits) to specify which *D* features.

If it is difficult to decide which encoding to use. One can always also mix the message lengths from different encodings. If $I_{1}$ and $I_{2}$ are message lengths of the same event encoded by two different coding schemes, then $I_{3}=-\ln\frac{e^{-I_{1}}+e^{-I_{2}}}{2}$ can be used as the message length of the event, and $I_{3}$ will be longer than $\min(I_{1},I_{2})$ by at most 1 bit (or ln2 nits):

(9) $$\begin{array}{c} \min\left(I_{1},I_{2}\right)\leq -\ln\frac{e^{-I_{1}}+e^{-I_{2}}}{2}<\min\left(I_{1},I_{2}\right)+\ln2 \end{array}$$

In this study, we will use the second method to encode the selection of features, i.e.,

(10) −lnhs(K,D)=ln(K+1)+lnKD

For a continuous prior, usually several priors are considered, e.g., the Jeffreys prior, Gaussian prior, or conjugate prior. The Jeffreys prior is generally advocated for being objective [91], though it is not really objective—more specifically, it favors parameter values where (the expected second derivative is large because) the measuring instruments are the most sensitive [92] (Section 2.3). It is also not recommended for multivariate models [93]. The reference prior [94] is recommended for multivariate models instead. However, the choice of reference prior entails a kind of model selection by requiring the parameters to be categorized into nuisance parameters and parameters of interest.

If we assume that the features are independent, the expected Fisher information matrix will be diagonal, and the continuous prior would be a product of the univariate Jeffreys priors for each parameter. This prior has been used in a previous MML study [95]. The issue is that in many cases the Jeffreys prior is improper [92] (Sections 2.3.2–2.3.4), and normalizing the product of multiple Jeffreys priors would often be computationally expensive.

A previous study [96] claims that there is no conjugate prior specification for the coefficients in the IPPP intensity function, and it is suggested using a Gaussian prior. We could use Gaussian priors with means equal to zero and large standard deviations to represent our ignorance about the parameters. In this study, we chose $h\left(\theta \right)∼N\left(0,10^{2}\right)$ as the prior distribution for each parameter $\theta_{i},i=1,2,\cdots ,D$ to represent our ignorance of parameter values, given that the features are standardized. The choice of σ=10 for this prior should lead to negligible bias, as discussed in Appendix A. The continuous prior can be written as follows:

(11) $$\;-\ln h\left(\theta \right)=\sum_{i=1}^{D}\left(\frac{1}{2}\ln\left(2\pi \right)+\ln\left(10\right)+\frac{\theta_{i}^{2}}{200}\right)$$

### 4.2. The Search Algorithm

To avoid searching for the best model by calculating the message length for all candidature models, we propose a search algorithm in this section. As mentioned in Section 4.1, the discrete prior we used (ln(K+1)+lnKD nits) prefer models with fewer or more features. Also from vague unproven experience, we speculate that the minimum message length usually shows a unimodal curve against the number of features. We could start the search with the initial number of features k=⌊D/2⌋, where *D* is the number of possible features. $I(M_{k})$ is the message length of model *M* with *k* features. The process is described in Algorithm 1:
**Algorithm 1** MML search algorithm.**Input:** Models *M***Output:** Best model $\hat{M}$1:k←D/22:$I\left({\hat{M}}_{k}\right)=min(I\left(M_{k}\right)$3:$I\left({\hat{M}}_{k-1}\right)=min\left(I\left(M_{k-1}\right)\right)$4:$I\left({\hat{M}}_{k+1}\right)=min\left(I\left(M_{k+1}\right)\right)$5:**if** $I\left({\hat{M}}_{k-1}\right)<I\left({\hat{M}}_{k+1}\right)$ **then**6:    dir=−17:**else**8:    dir=19:**end if**10:**while** $I\left({\hat{M}}_{k+dir}\right)<I\left({\hat{M}}_{k}\right)$ **do**11:    k←k+dir12:**end while**13:**return** $\hat{M}\leftarrow {\hat{M}}_{k}$

## 5. Simulations

In this section, we conduct simulations for two scenarios: feature selection given the correct model family and model selection with model family misspecification, including model families with aggregated data.

### 5.1. Simulation 1: Feature Selection

To simulate a feature selection scenario, we constructed a simulation to compare the capabilities of MML, AIC, AICc, and BIC in identifying the relevant features (as they are all information-based criteria, MML has been compared with the other three commonly used methods). The simulation was constructed (in terms of LNPPP space [29] (Section 0.2.7), and [54] (Section 6.1)) as follows: the features were extracted from a real-world dataset collected on Barro Colorado Island (BCI) [97]. The dataset contains measures of 13 nutrient elements in the soil in a divided 1000×500 $m^{2}$ region, with each grid covering a 20×20 $m^{2}$ rectangle.

**Figure 1:**
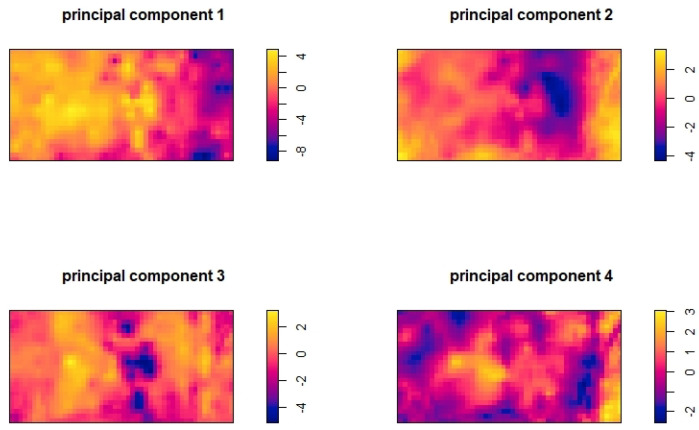
The distributions of the 4 principal components across the survey area.

**Figure 2:**
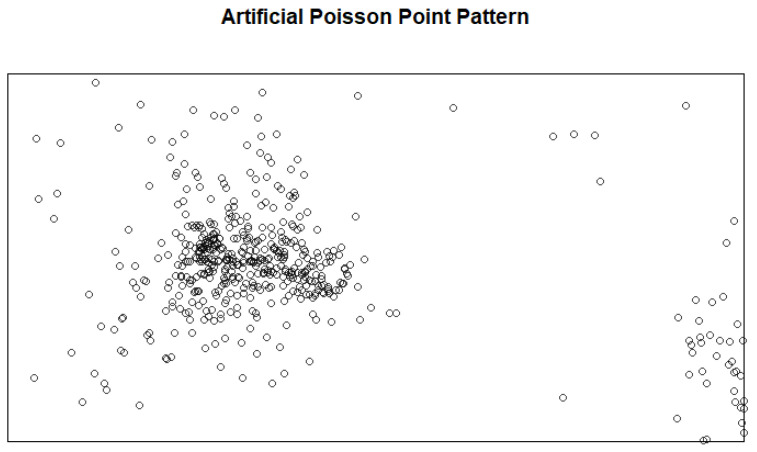
A Poisson point pattern randomly generated by the artificial intensity function and the 4 principal components.

We first standardized each feature and then performed a principal component analysis (PCA) to guarantee independent features. The first 12 principal components were included in the simulations. The artificial intensity function λ(u) is defined as follows:

(12) $$\ln\lambda (u)=\frac{PC_{1}}{\sigma_{1}}+\frac{PC_{2}}{\sigma_{2}}+\frac{PC_{3}}{\sigma_{3}}+\frac{PC_{4}}{\sigma_{4}}-3$$

where $PC_{1},PC_{2},PC_{3},PC_{4}$ are the first four principal components, and the same number of features has been used in a previous study [98] for feature selection in the IPPP. The distributions of these four principal components are shown in Figure 1. As the principal components have a descending order of contributions to the variance of the data, to balance the contribution of each principal component to the intensity function, each principal component was weighted by the inverse of its standard deviation, i.e., $1/\sigma_{1},1/\sigma_{2},1/\sigma_{3},$ and $1/\sigma_{4}$, respectively. The simulated Poisson point patterns were then generated using this intensity function. Figure 2 shows one of these generated Poisson point patterns.

In practice, two main feature selection challenges in ecological studies include the following: (1) the sample size can be relatively small; and (2) there are features irrelevant to the species distribution. In this simulation, each test had the first 8, 10, or 12 principal components as possible features to simulate the situation where irrelevant features exist. In addition, to simulate relatively small, moderate, and large sample sizes, we applied a random thinning process to the generated Poisson point patterns. The thinning process randomly removes points in the Poisson point patterns with a fixed probability *p*. This process will retain the Poisson property of the generated Poisson point patterns. In this test, each simulation will have 1 (no thinning), 0.75, (three-quarters retained), or 0.5 (half retained) as the thinning probability.

To measure the accuracy of identifying the true subset of features, i.e., the similarity between the selected subset of features and the true features being used to generate the artificial intensity function, we define the generalized Hamming distance *d* between a set of features $X=\{X_{1},X_{2},\ldots ,X_{n}\}$ and the set $Y=\{PC_{1},PC_{2},PC_{3},PC_{4}\}$ as follows:

(13) d=|N(X)−N(X∩Y)|+|N(Y)−N(X∩Y)|

where *N* is the number of elements in a given set. In simulations, the smaller *d* represents the selected subset more similar to *Y*, while d=0 means that the feature selection criterion correctly selects the true subset of features.

For each test with the given possible features and thinning probability, we ran 50 trials. In each trial, a random Poisson point pattern was generated from the artificial intensity function. Then, every subset (except the empty set) of the possible features was used to generate models to fit the data, e.g., with eight possible features, there are 255 subsets except the empty set; hence, a total of 255 models are generated and fitted to the data. For each model, all parameters were estimated using the ppm() function from the spatstat package [27] in R. The AIC, AICc, and BIC methods then selected the best models according to their criteria, while the MML method used the search algorithm to find the model with minimum message length. The AIC selects the model that minimizes AIC=−2l+2k, where *l* is the log-likelihood, and *k* is the number of features used in the model. The AICc selects the model that minimizes $AICc=-2l+2k+\frac{2k(k+1)}{n-k-1}$, where *n* is the number of observations. The BIC selects the model that minimizes BIC=−2l+klnn. A new Poisson point pattern was then generated with the same intensity function for testing purposes. The selected models were used for predictions on the test Poisson point pattern. Then, the models selected by MML, AIC, AICc, and BIC methods were compared based on two metrics: the average generalized Hamming distance *d* of 50 trials and the average log-likelihood of their predictions on the new Poisson point pattern, i.e., the negative log-loss/binary cross-entropy of their predictions. We briefly digress and note that, in general, precision and recall (also known as sensitivity) and (their harmonic mean) F1-score do not remain invariant under (e.g.) the simple transformation of interchanging the positive and negative classes. However, log-loss (or logarithm loss) is invariant under this transformation and also under far more general transformations than (simply) interchanging positive and negative classes. Further justification for using log-loss as a measure of prediction power (including its uniquely being invariant under general transformations) is discussed in various places [29] (ftn 175 and 176), [99] (pp. 437–438), [52] (Section 3), [100] (Section 4.1), and [54] (Section 6).

### 5.2. Simulation 1: Feature Selection—Results and Discussion

From Table 1, we can see that in all nine tests, MML consistently had an average generalized Hamming distance (*d*) equal to 0. This indicates that MML accurately identifies the true subsets of features in all trials. In addition, in all tests, MML consistently outperformed other criteria in both the feature selection accuracy and prediction performance.

In particular, MML outperformed the AIC and AICc in identifying relevant features that have significant impacts on the target variable. Additionally, when the dataset had a smaller sample size and a larger number of features, MML surpassed the BIC. Furthermore, when there was both a large number of irrelevant features and (at the same time) a small sample size, MML exhibited significantly higher robustness when compared to AIC, AICc, and BIC.

One thing to note is that we used Maximum Likelihood estimation (MLE) for point estimations and then selected models based on these estimations. This strategy likely afforded the AIC and BIC advantages, as they are developed based on MLE. We chose this strategy instead of using another estimator to find the point estimation that minimizes the message length because, first, using the same estimator for all model selection methods ensures consistency in comparisons; this strategy was also used in [12,13]. Second, to our knowledge, there is no reliable way to find the point estimation that minimizes the message length of an IPPP. Third, this paper focuses on model selection techniques, and point estimation is beyond its scope.

### 5.3. Simulation 2: Model Family Misspecification

**Figure 3:**
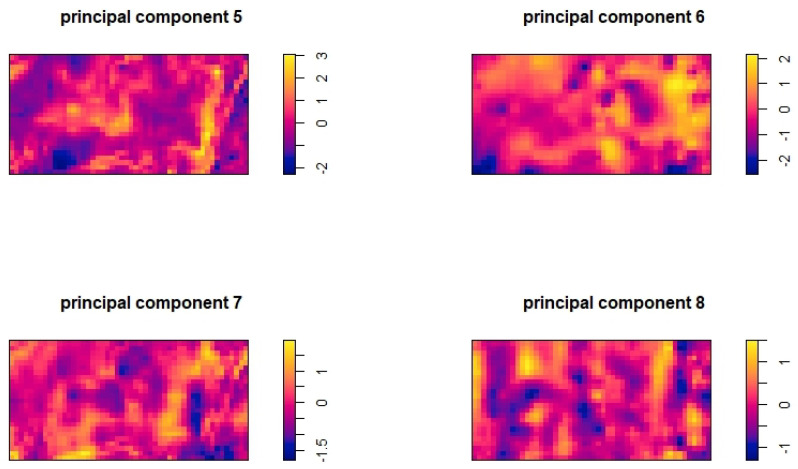
The distributions of another 4 principal components across the survey area.

**Figure 4:**
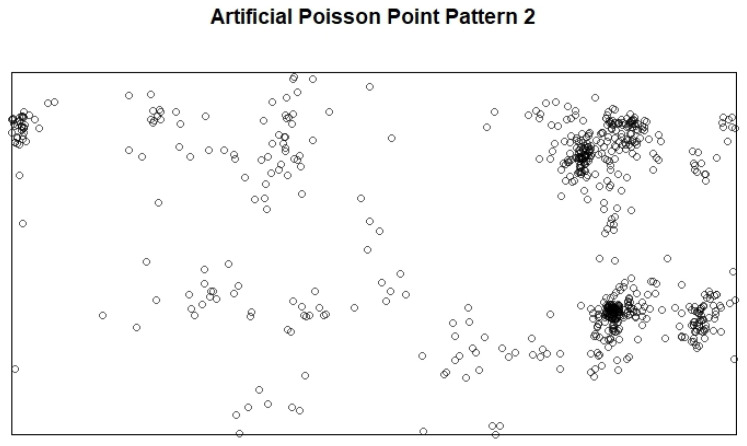
A Poisson point pattern randomly generated by the artificial intensity function and the 5th to 8th principal components.

To simulate a model family misspecification scenario and test the robustness of model selection criteria, we conducted a simulation with a configuration similar to the test in Section 5.1. We used the fifth to eighth principal components for data generation this time. The artificial intensity function λ(u) we used is defined as follows:

(14) $$\begin{array}{c} \ln\lambda (u)=\frac{PC_{5}}{\sigma_{5}}+\frac{PC_{6}}{\sigma_{6}}+\frac{PC_{7}}{\sigma_{7}}+\frac{PC_{8}}{\sigma_{8}}-3 \end{array}$$

where $PC_{5},PC_{6},PC_{7},$ and $PC_{8}$ are the four principal components, and $\sigma_{5},\sigma_{6},\sigma_{7},$ and $\sigma_{8}$ are their standard deviations, respectively. The distributions of these four principal components are shown in Figure 3. An example of the Poisson point patterns generated is shown in Figure 4.

To measure the robustness of the MML model selection criterion against model family misspecification given multiple model families, we used the Poisson distribution and the negative binomial distribution as candidature model families. The negative binomial distribution was used to model count data with overdispersion, where the variance exceeds the mean. Also, since in practice, sometimes we might want to find another perspective to explore the relationship of the data, we aggregated the data and transfered them to another format. We used logistic regression as a candidate model family to simulate the scenario of data aggregation. The logistic regression is often used for occupancy analysis, so we needed to convert the count data to occurrence data (a form of binary data, with 1 representing a grid being occupied and 0 otherwise). The application of MML to the estimation of Poisson distributions has also been studied [73,75]. MML model selection with logistic regressions has been discussed previously [16] as well.

We used the Wallace-Freeman MML87 formula described in Section 4 to calculate the message length of each model. For a Poisson distribution, usually a Gamma prior is considered, as it is a conjugate prior. For logistic regression, it is suggested to use Cauchy priors or Gaussian priors [101]. Since the main purpose of this simulation was to compare the robustness of model selection criteria, not parameter estimation (this part is done by the glm() function in R), a conjugate prior was not necessary here. To maintain consistency, we used the Gaussian prior used in Section 4.1 as the prior for all parameters in Poisson distributions and logistic regressions. For the negative binomial distribution, we used the glm.nb() function in R for parameter estimation, which returns not only the estimated parameters but also a dispersion measure θ. We used the same Gaussian priors for the parameter estimations. The dispersion measure θ was estimated according to [102] (pp. 186, 208):

(15) $$\;\theta =\frac{D_{M}}{n-p}$$

where $D_{M}$ is the residual sum of squares, and n−p is the residual degrees of freedom. This measure cannot be less than 0, so a Gaussian prior is inappropriate. Since we have 1250 data points with at most eight features, the residual degrees of freedom will not be less than 1241, and it is very likely that θ will not be large. We could use a negative exponential distribution as the prior for θ, with a mean μ equal to the mean of the count data. The negative exponential prior has also been used in MML work before [12,13].

To conduct the simulation, we randomly generated a Poisson point pattern from Equation (14), and then we generated the count data and the occurrence data by converting the point pattern. This can be easily done with logistic regressions by turning the point pattern to occurrence data, i.e., if a grid has observations, then it is occupied. For Poisson distributions and negative binomial distributions, we needed to convert the point pattern into count data by counting the number of observations for each grid. We then fit the Poisson distributions and the negative binomial distributions to the count data with all possible combinations of the first eight principal components. For each combination, we used the glm() function (for the Poisson distribution and the logistic regression) and the glm.nb() function (for the negative binomial distribution) in R to obtain the parameter estimations. Then we used the AIC, AICc, and BIC to select the best models while using the search algorithm to find the model with the minimum message length. After finding the best models, we generated a new Poisson point pattern from the intensity function, converting it to count data and occurrence data again, and calculated the log-likelihood of the selected models on the new point pattern as a measure of prediction performance. This process was repeated 50 times. We also fit the logistic regressions to the occurrence data 50 times with the same configuration. We again used the generalized Hamming distance to measure the accuracy of feature selection.

### 5.4. Results and Discussion—Model Family Misspecification

As seen in Table 2, in all 50 random trials, all criteria selected negative binomial distributions between negative binomial distributions and Poisson distributions. This indicates that there is an overdispersion in the count data. Thus, Poisson distributions are inappropriate for the data.

In all 50 trials, MML showed superior performance in feature selection with an average Hamming distance equal to 0. This means that MML correctly selected the feature used to generate the point patterns, even when the model was misspecified. This result matches the previous study [12,13], where MML also showed a surprisingly strong robustness against model misspecification compared to other criteria. The prediction performances of the models selected by the different criteria (AIC, AICc, BIC, MML) did not have significant differences from one another.

Table 3 shows the performance of the model selection of different criteria against not only model misspecification but also data aggregation. The prediction performances barely varied and had no significant differences. MML still held the smallest average Hamming distance, while the AIC and AICc selected the same models again, and the BIC selected features better than AIC and AICc.

In summary, MML showed superior performance as a model selection criterion in all 50 trials under different scenarios. Compared to the AIC, AICc, and BIC, MML had the most robust model selection performance when the models were from multiple incorrect model families. MML not only correctly selected the better model family but also correctly selected the features that generated the data in all trials, while no other criteria achieved the same level of correctness. Moreover, even when fed with aggregated data and a misspecified model family, MML still correctly selected the features in most of the trials and outperformed other criteria. Overall, MML has a surprising robustness against model misspecification and data aggregation compared to the AIC, AICc, and BIC.

## 6. An Ecological Case

**Figure 5:**
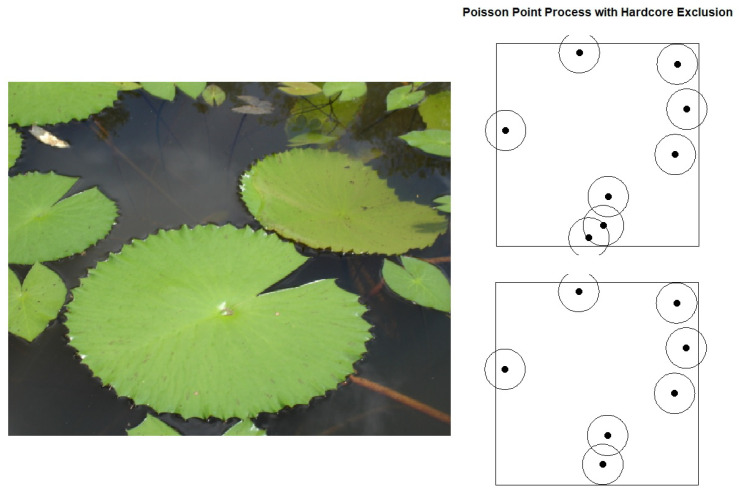
The *Nymphaea lotus* leaves [105] and the hardcore process. The two points at the bottom have their disc overlapped; thus, one is randomly removed.

We mentioned the IPPP in Section 4 and used it to generate simulated point patterns in Section 5. The IPPP assumes that the individuals are independently distributed from each other in the survey area. However, for some species, this is not true, and we would not use the IPPP to describe their distributions. The hardcore process is one of the point processes that models the interactions between points. It is a special case of the Neyman–Scott process [103]. The hardcore process is a two-step model. In the first step, a parent process (usually the IPPP) generates points in the study area. Then, following the rule that for each point, no other points exist within a certain distance, points that have their exclusive zones overlapped with other zones are removed until no zones overlap. The hardcore process creates a repulsive effect, resulting in a spatial pattern where points tend to be more regularly spaced [104]. This repulsive effect might be caused by several behaviors, including resource competition and territoriality. An example would be lotus leaves. The distributions of lotus leaves are hardcore-like (not exactly a hardcore process, since the radius of a leaf is not fixed). Since a lotus leaf will cover all the seeds under it within its shadow, these seeds will not gain enough sunlight. This is a very classic resource competition case. The hardcore process is shown in Figure 5. When two points violate the exclusion zones of each other, one of them is randomly selected and removed (other removing rules might apply depending on the context, and here we use the simplest one by totally randomly selecting one to remove).

### 6.1. Barro Colorado Island Data Preprocessing and Experiment Protocol

We considered the tropical forest survey data collected within a 50-hectare (ha) area on Barro Colorado Island (BCI), Panama [106,107]. The Barro Colorado Forest census plot data (or BCI 50 ha plot data) is an accurate, comprehensive dataset of point records for every individual tree in the 50 ha plot. It was established in 1980 in the tropical moist forest of BCI in Gatun Lake in central Panama. The dataset contains the spatial distributions of 455 species across the island, with 20 cm used as the precision of the coordinates.

In this experiment, we used the IPPP and the hardcore process to fit the data. Since collinearity could exist in the data, and PCA is one of the methods recommended to handle collinearity by the previous study [108], we also included the IPPP with PCA data as one of the candidate model families.

With every possible subset except the empty set of the environmental features in the BCI soil data (used in Section 5), we fit the IPPP and hardcore process to the distribution of two species, namely, *Cassipourea elliptica* and *Calophyllum longifolium*. We also did the same with every possible subset except the empty set of the principal components of the environmental features. All parameter values were estimated by the ppm() function in R. The AIC, AICc, and BIC were then used to select the best model from the IPPP, the hardcore process, and the IPPP with PCA data, while the search algorithm was used to find the model with the minimum message length. A 10-fold block cross-validation was used to generate the training sets and corresponding test sets for evaluation purposes. Again, the log-likelihood of the predictions made by the models selected by different criteria was used to measure the prediction performance of the criteria.

The estimated hardcore process has a set of features similar to the IPPP, except that it also has a parameter radius *r*. *r* must be larger than 0 (it becomes an IPPP when r=0). We would expect that as *r* increases, the probability density of *r* becomes smaller, since the coverage grows faster than the radius, and it is unlikely that a single individual covers an infinitely large amount of resource. Similarly to the dispersion measure in the negative binomial distribution case in Section 5.3, we used a negative exponential prior h(μ) for *r*. A vague guess of the hyperparameter μ for this prior could be as follows:

(16) $$\mu =\sqrt{|A|/n}$$

where |A| is the size of the survey area, and *n* is the number of observations. This is the edge length of the squares when assuming that each point occupies a square uniformly instead of a disc. With this prior on *r*, the message length of the hardcore process becomes the following:

(17) $$\;\begin{array}{cc} I= & -\ln\pi \left(\theta \right)-\ln h\left(\mu \right)+\frac{1}{2}\ln\left|F\left(\theta ,\mu \right)\right|\\ \; & +\frac{D+1}{2}\ln\kappa_{D+1}-\ln f\left(Y|\theta ,\mu \right)+\frac{D+1}{2} \end{array}$$

### 6.2. Results and Discussion

**Figure 6:**
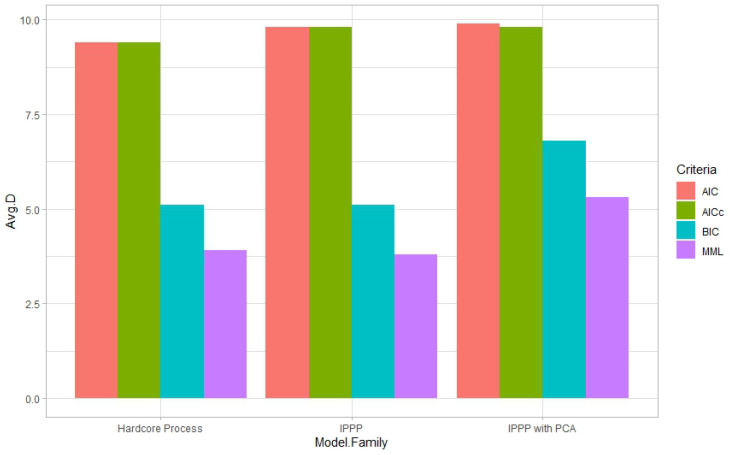
Average number of features selected from three model families in the block cross-validation by different criteria for *Cassipourea elliptica*.

**Figure 7:**
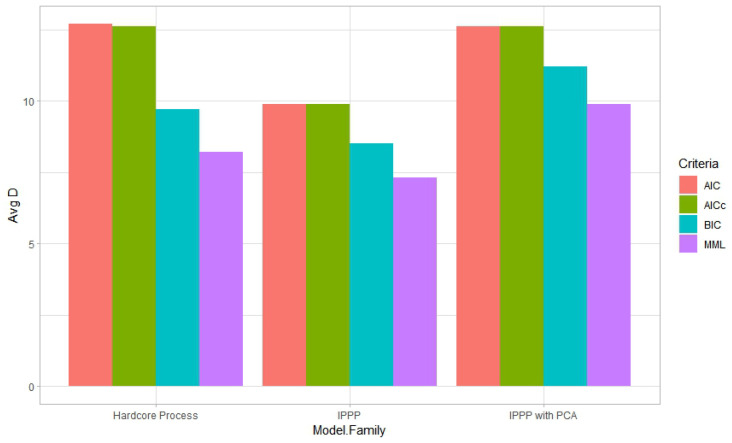
Average number of features selected from three model families in the block cross-validation by different criteria for *Calophyllum longifolium*.

From Table 4 and Table 5, we can see that MML selected the fewest features when specifically compared to the AIC and AICc. Again, the predictions did not have significant differences. All criteria selected the hardcore process as the best model family. However, for both species, MML and BIC selected the fewest features while also selecting the IPPP as the second-best model family, while the AIC and AICc favored the IPPP with PCA data over the IPPP. This might have occurred, since every principal component is a combination of all the features. Using PCA data means that none of the original features can be excluded from the model. MML and BIC tended to select simpler models, as we can see from Figure 6 and Figure 7. Thus, instead of selecting the IPPP with PCA data, which means involving all features in modeling, MML and BIC preferred the IPPP without PCA data, even if collinearity might exist in the raw data.

Although there is no ecological evidence that the two plants have the repulsive effect or the habit of growing exclusively in space, due to the nature of species distribution data, it is normal to find that the hardcore process fits the data better than the IPPP. Since we only have a limited number of observations, and usually this amount is not large due to the expensive cost of conducting field surveys, we would expect that there is a certain minimum distance between points existing in the data. Specifying that minimum distance in the model should explain at least some of the variance of the data points, and thus lead to better fitness.

## 7. Conclusions

This paper proposed the minimum message length (MML) framework for model selection from multiple model families in species distribution modeling (SDM). The calculation of the message length of point processes with the log-linear intensity function is provided, and the choice of the Bayesian priors is discussed. We also proposed a search algorithm used in MML model selection that can avoid brute-force search and still maintain a good accuracy of model selection.

The simulation results on artificial data show that in nine tests where the correct model family is given, MML as a feature selection criterion always correctly selected the relevant subset of features, while the competing criteria all failed on a variety of tests. And in a model family misspecification test, MML still correctly selected the relevant subset of features, while the other criteria failed to do so. Even when the data were aggregated and the model family was misspecified, MML still selected the subset of features that was closest to the correct subset of features, being closer than all the alternative methods.

The real-world experiments demonstrate that for each of the two plant species, MML selected the simplest models, and the models selected by MML had the overall best predictions.

This suggests that practitioners and managers in conservation planning can use MML to reliably select the best model family and identify relevant features. After preprocessing the data and preparing candidate model families, practitioners can use the search algorithm to find the model with the minimum message length, and they can then can identify the best model family and feature subset. The result is expected to be overall better than the results obtained by the AIC, AICc, and BIC. 

## Figures and Tables

**Table 1:** K is the number of possible features used for modeling, and *p* is the thinning probability. d is the average generalized Hamming distance between the selected subset of features and the set of relevant features. logLik is the average log-likelihood, and *t*-test *p*-value is the *p*-value of the *t*-test between the numbers of features selected by MML and by other feature selection criteria. In MML calculation, the parameters each have $N(0,10^{2})$ Bayesian priors, and the selection has a Bayesian prior explained in Formula (10).

	MML		AIC			AICc			BIC		
	d	logLik	d	logLik	*t*-test *p*-value	d	logLik	*t*-test *p*-value	d	logLik	*t*-test *p*-value
K = 8, *p* = 0.5	**0.00**	**−225.89**	0.72	−226.82	<0.01	0.68	−226.77	<0.01	0.08	−226.14	0.04
K = 8, *p* = 0.75	**0.00**	**−183.77**	0.60	−184.60	<0.01	0.58	−184.61	<0.01	0.04	−183.93	0.16
K = 8, *p* = 1	**0.00**	**−91.88**	0.50	−92.77	<0.01	0.46	−92.66	<0.01	0.02	−91.90	0.32
K = 10, *p* = 0.5	**0.00**	**−227.88**	1.12	−229.90	<0.01	0.90	−229.61	<0.01	0.08	−228.32	0.04
K = 10, *p* = 0.75	**0.00**	**−186.87**	1.10	−188.58	<0.01	1.10	−188.58	<0.01	0.04	−186.88	0.32
K = 10, *p* = 1	**0.00**	**−80.04**	1.08	−82.22	<0.01	1.08	−82.22	<0.01	0.06	−80.22	0.08
K = 12, *p* = 0.5	**0.00**	**−224.29**	1.10	−226.80	<0.01	1.04	−226.55	<0.01	0.26	−225.45	<0.01
K = 12, *p* = 0.75	**0.00**	**−180.55**	1.38	−182.47	<0.01	1.3	−182.42	<0.01	0.16	−181.42	0.01
K = 12, *p* = 1	**0.00**	**−98.34**	1.42	−101.37	<0.01	1.38	−101.35	<0.01	0.18	−99.21	<0.01

**Table 2:** Simulation of model selection with multiple model families (i.e., Poisson distributions and negative binomial distributions) against model misspecification. d is the average Hamming distance. logLik is the average log-likelihood of the selected models on the new data. *t*-test *p*-value is obtained by comparing the log-likelihood of the models selected by MML with the log-likelihood of models selected by the criterion, with the null hypothesis that no significant difference exists. model is the model family selected by the criterion, with NB being negative binomial distributions while PO being Poisson distributions. In MML calculation, the parameters each have $N(0,10^{2})$ Bayesian priors, and the selection has a Bayesian prior explained in Formula (10).

	MML	AIC	AICc	BIC
d	**0**	1.6	1.6	0.28
logLik	−672.601	**−671.887**	**−671.887**	−672.940
*t*-test *p*-value	N/A	0.8421	0.8421	0.9243
model	NB	NB	NB	NB

**Table 3:** Simulation of model selection against model misspecification and data aggregation. d is the average Hamming distance. logLik is the log-likelihood of the selected model on the new data. *t*-test *p*-value is obtained by comparing the log-likelihood of models selected by MML with the log-likelihood of models selected by the criterion, with the null hypothesis being no significant difference exists. In MML calculation, the parameters each have $N(0,10^{2})$ Bayesian priors, and the selection has a Bayesian prior explained in Formula (10).

	MML	AIC	AICc	BIC
d	**0.08**	1.32	1.32	0.3
logLik	**−369.336**	−369.493	−369.493	−369.559
*t*-test *p*-value	N/A	0.9642	0.9642	0.9497

**Table 4:** Results of model selection from multiple model families for *Cassipourea elliptica*. D is the number of features selected, and loglik is the log-likelihood. *t*-test *p*-value is the *p*-value of the paired *t*-test between the log-likelihood on the test set of models selected by MML and by other criteria given the null hypothesis that their means are equal. CM is the criterion measure corresponding to the measure of each criterion, i.e., Avg.CM is *x* for MML means the average message length of the models selected by MML is *x*. In MML calculation, the parameters each have $N(0,10^{2})$ Bayesian priors, and the selection has a Bayesian prior explained in Formula (10). The hardcore process has an extra parameter μ that follows a negative exponential distribution, which is explained in Formulas (16) and (17).

		MML	AIC	AICc	BIC
Inhomogeneous Poisson Point Process	Avg.D	3.8	9.8	9.8	5.1
	Avg.loglik	−972.678	−981.643	−981.643	−979.141
	*t*-test *p*	N/A	0.962	0.962	0.972
	Avg.CM	8637.255	17,183.97	17,187.37	17,218.74
Hardcore Process	Avg.D	3.9	9.4	9.4	5.1
	Avg.loglik	−972.949	−979.821	−979.821	−979.279
	*t*-test *p*	N/A	0.970	0.970	0.973
	Avg.CM	8629.565	17,169.47	17,169.66	17,210.47
Inhomogeneous Poisson Point Process with PCA Data	Avg.D	5.3	9.9	9.8	6.8
	Avg.loglik	−968.662	−981.526	−981.506	−976.560
	*t*-test *p*	N/A	0.988	0.965	0.964
	Avg.CM	8644.475	17,182.73	17,187.16	17,223.51

**Table 5:** Results of model selection from multiple model families for *Calophyllum longifolium*. D is the number of features selected, and loglik is the log-likelihood. *t*-test *p*-value is the *p*-value of the paired *t*-test between the log-likelihood on the test set of models selected by MML and by other criteria given the null hypothesis that their means are equal. CM is the criterion measure corresponding to the measure of each criterion, i.e., Avg.CM is *x* for MML means the average message length of the models selected by MML is *x*. In MML calculation, the parameters each have $N(0,10^{2})$ Bayesian priors, and the selection has a Bayesian prior explained in Formula (10). The hardcore process has an extra parameter μ that follows a negative exponential distribution, which is explained in Formulas (16) and (17).

		MML	AIC	AICc	BIC
Inhomogeneous Poisson Point Process	Avg.D	8.2	12.7	12.6	9.7
	Avg.loglik	−1596.682	−1616.531	−1615.957	−1612.654
	*t*-test *p*	N/A	0.963	0.964	0.970
	Avg.CM	13,917.81	27,679.83	27,686.17	27,741.78
Hardcore Process	Avg.D	7.3	9.9	9.9	8.5
	Avg.loglik	−1598.482	−1611.484	−1611.484	−1606.084
	*t*-test *p*	N/A	0.976	0.976	0.986
	Avg.CM	13,892.15	27,653.55	27,653.66	27,711.59
Inhomogeneous Poisson Point Process with PCA Data	Avg.D	9.9	12.6	12.6	11.2
	Avg.loglik	−1624.481	−1615.368	−1615.368	−1610.446
	*t*-test *p*	N/A	0.983	0.983	0.974
	Avg.CM	13,920.75	27,675.53	27,680.78	27,746.19

**Figure A1:**
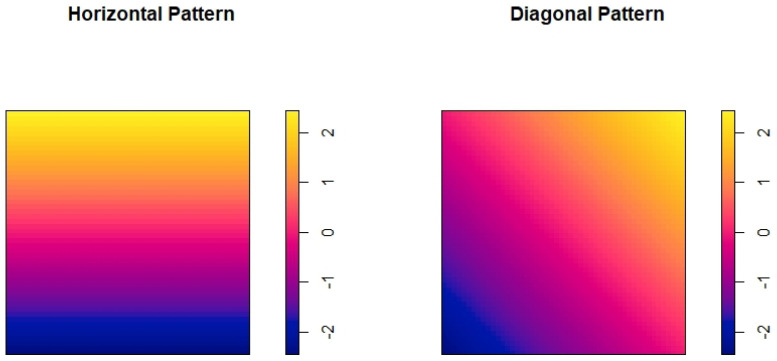
Horizontal pattern and diagonal pattern.

**Figure A2:**
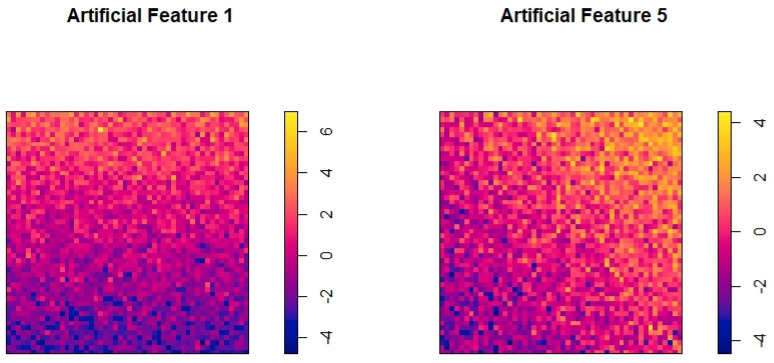
Artificial features 1 and 5.

**Figure A3:**
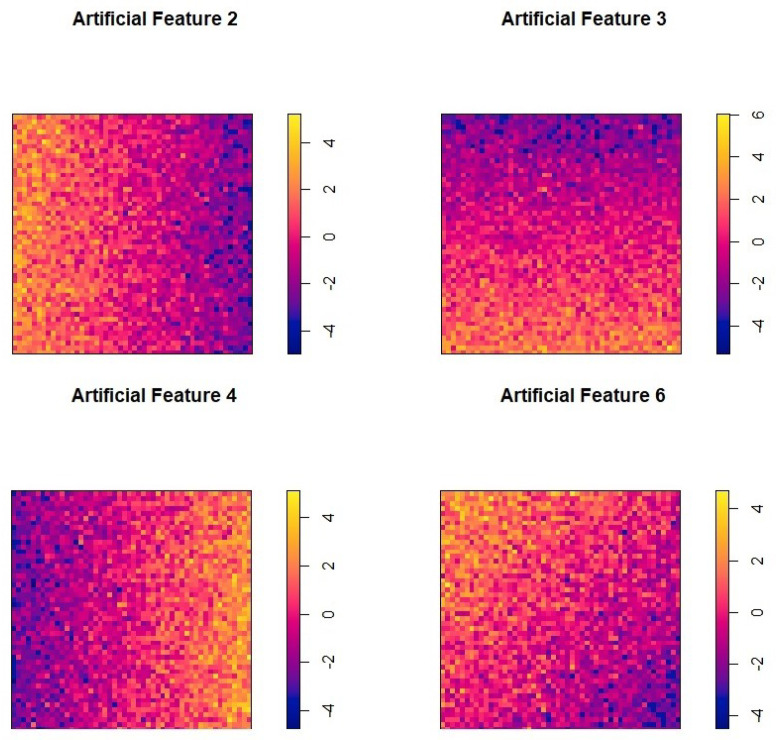

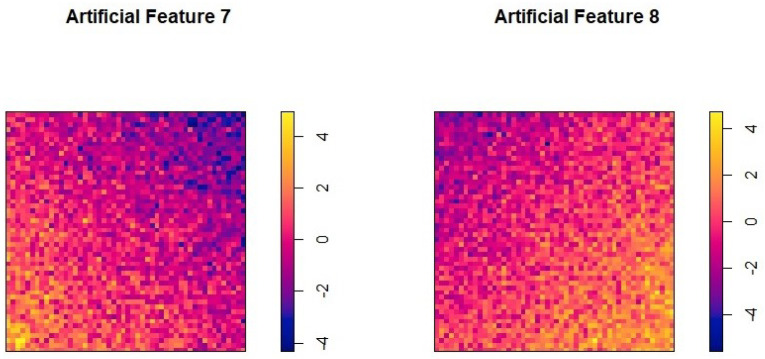
Artificial features 2, 3, 4, 6, 7, 8.

**Figure A4:**
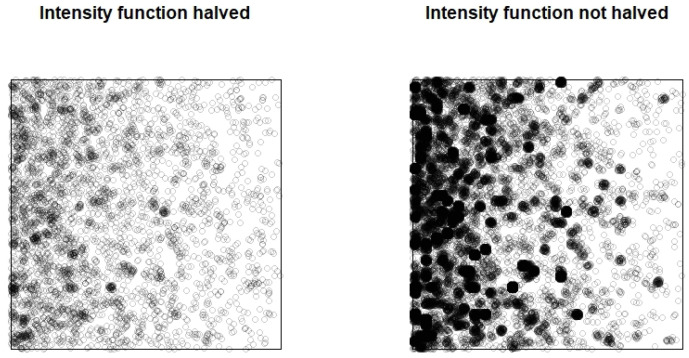
Examples of Poisson point patterns generated with and without intensity being halved.

**Figure A5:**
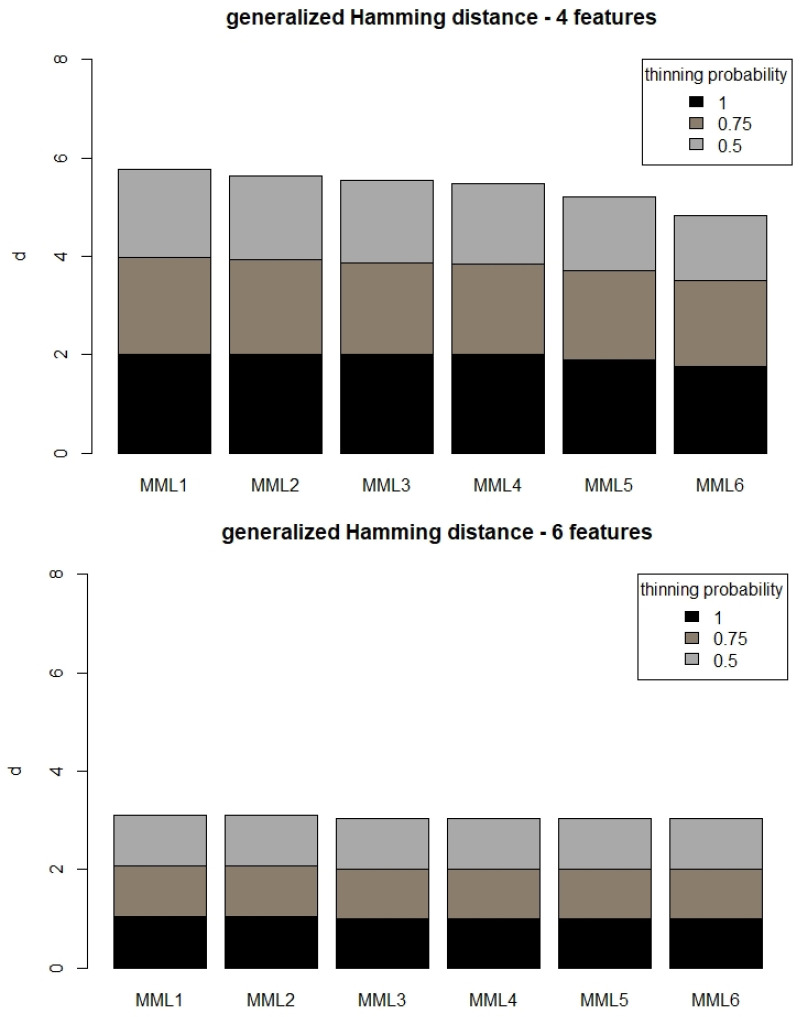

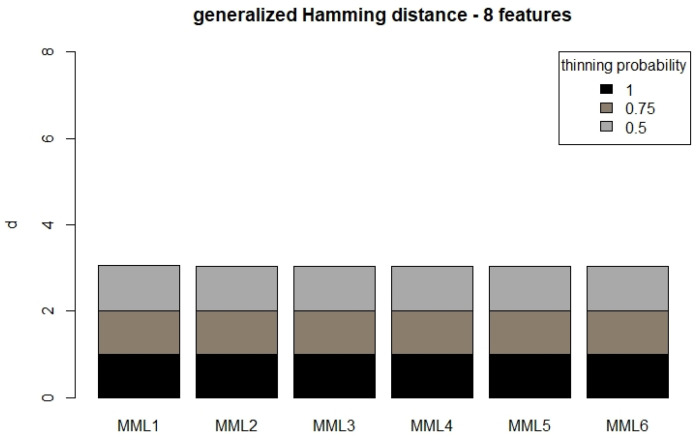
Generalized Hamming distance with 4, 6, and 8 features given for modeling. MML1 to MML6 are MML metrics with six different Gaussian priors; each has zero mean and variance equal to $1^{2},2^{2},5^{2},10^{2},20^{2},100^{2}$, respectively.

**Figure A6:**
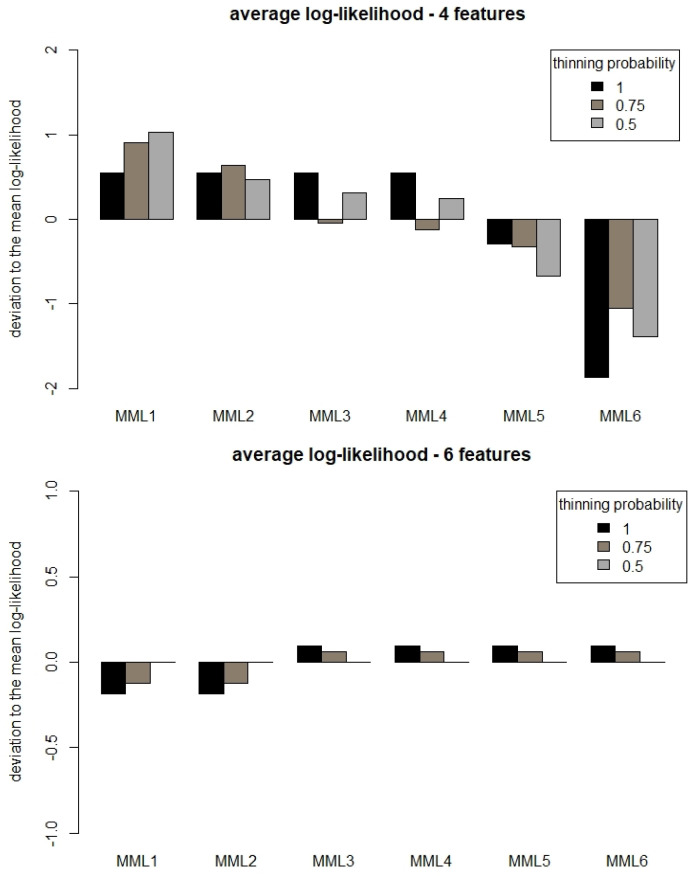

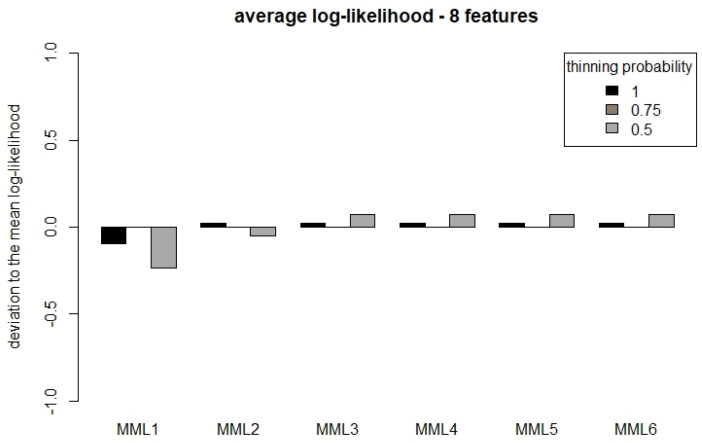
Average log-likelihood for predictions with 4, 6, and 8 features given for modeling. Note that the differences are too small compared to the log-likelihood values (e.g., the mean log-likelihood for 4 features with thinning probability equal to 1 is −1074.822, while the deviation of different metrics is within [−2, 2]). MML1 to MML6 are MML metrics with 6 different Gaussian priors; each has zero mean and variance equal to $1^{2},2^{2},5^{2},10^{2},20^{2},100^{2}$ respectively.

**Figure A7:**
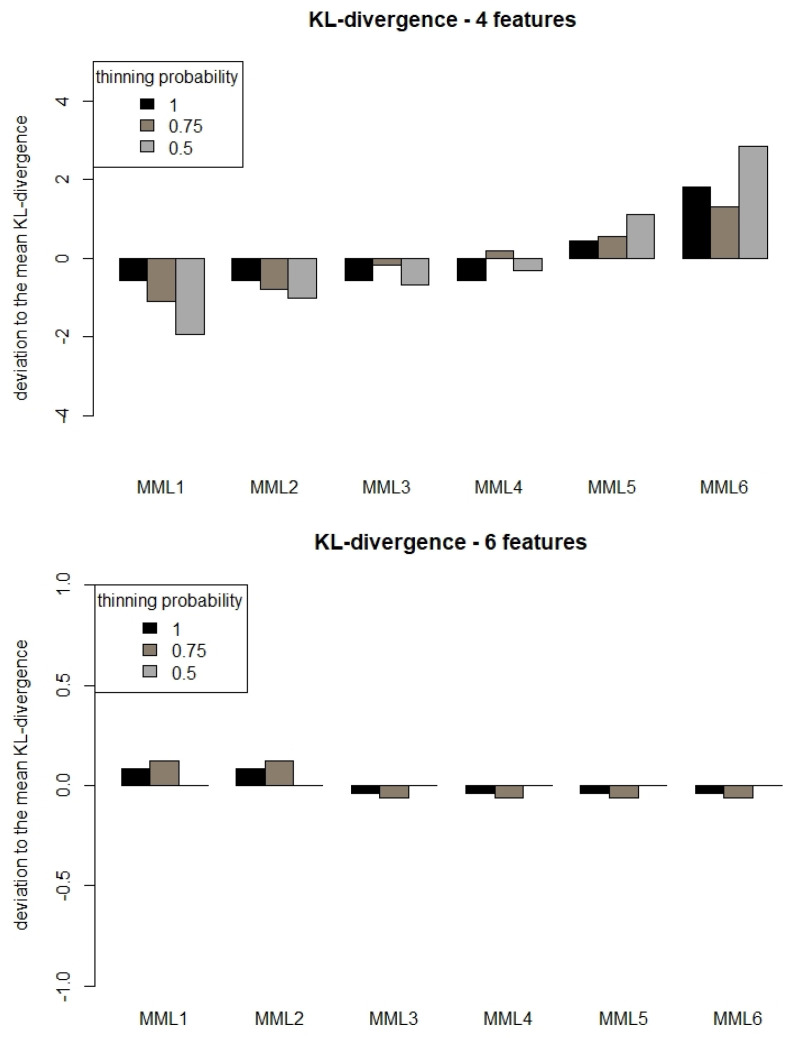

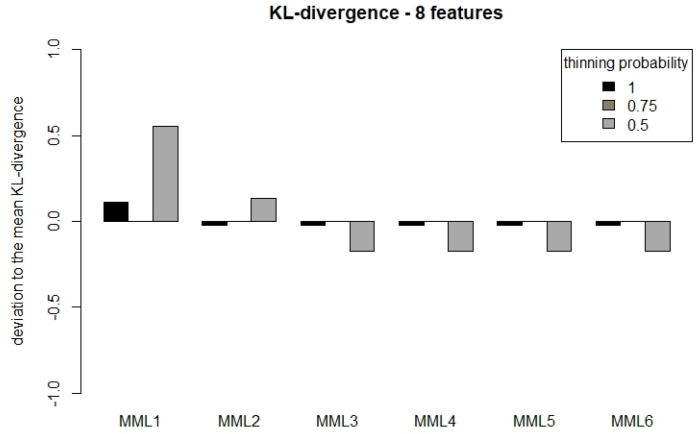
KL divergence with 4, 6, and 8 features given for modeling. Note that the differences are too small compared to the KL divergence values (e.g., the mean KL divergence for 4 features with thinning probability equal to 1 is 899.885, while the deviation of different metrics is within [−5, 5]). MML1 to MML6 are MML metrics with 6 different Gaussian priors; each has zero mean and variance equal to $1^{2},2^{2},5^{2},10^{2},20^{2},100^{2}$, respectively.

## Data Availability

The data presented in this study are available on request from the corresponding author.

## Abbreviations

The following abbreviations are used in this manuscript:

SDM	Species distribution modeling
BCI	Barro Colorado Island
AIC	Akaike information criterion
BIC	Bayesian information criterion
KL	Kullback–Leibler
KL Divergence	Kullback–Leibler divergence
MML	Minimum message length
IPPP	Inhomogeneous Poisson point process
PCA	Principal component analysis
AICc	Finite-sample corrected Akaike information criterion
MLE	Maximum likelihood estimation

## Appendix A. Justification of the Bayesian Prior Selected

Gaussian priors with large variances are sometimes referred to as weakly informative priors. As discussed in Section 3, we used Gaussian priors for all parameter estimations in the IPPP. To discuss how large the variance should be so that the priors could be considered “weakly informative”, we constructed nine simulations.

Instead of using real-world feature data, we generated some artificial data so that we could run simulations with different data types. A horizontal pattern and a diagonal pattern were generated in a 50 × 50 area, respectively (shown in Figure A1). The horizontal pattern was generated as follows: for the pixel in the *i*th row and the *j*th column, its value was set as i/10−2.55. And in the diagonal pattern, the pixel in the *i*th row and *j*th column had its value as (i+j)/20−2.55. The constant 2.55 was the mean value of the patterns, and it was removed to keep the patterns with means equal to zero, so when using these patterns to generate intensity functions, the lambda would not be too large.

Then, distributions of feature 1 and feature 5 were generated by adding a N(0,1) noise to each pattern (shown in Figure A2). The distributions of features 2, 3, and 4 were generated by counterclockwise rotation of the horizontal pattern by 90 degrees every time and then adding an N(0,1) noise, respectively. The distributions of features 6, 7, and 8 were generated by counterclockwise rotation of the diagonal pattern and adding the same noise, respectively (shown in Figure A3).

After generating the distributions of the artificial features, the intensity function λ(u) is defined as follows:

(A1) $$\ln\lambda (u)=\frac{1}{2}\left(F_{1}+F_{3}+F_{6}+F_{7}\right)$$

The intensity being halved is to prevent too many points from overlapping with each other in the generated Poisson point patterns, as shown in Figure A4.

### Appendix A.1. Justification of Bayesian Prior-Simulations

Given the distributions of artificial features and the intensity function, we could now generate the Poisson point patterns. The intensity function contained features 1, 3, 6, and 7. In the simulations, the first 4, 6, and 8 features were used for modeling in our case to simulate different scenarios (e.g., with only the first four features being used for modeling, the simulation is simulating the scenario where both correlated and uncorrelated features are used in modeling, and some other correlated features are missing). Also since in practice the abundance of observations varies, we thinned the generated Poisson point patterns to simulate the rare/normal/abundant observations. An IPPP can be generated by applying thinning probabilities to each point [109]. In this test, since we wanted to maintain the parameterization, we applied a fixed thinning probability *k* to each point, that is, each point had a probability of *k* to be removed. We used 1, 0.75, and 0.5 as the *k* values for each scenario. We generated nine combinations of different thinning probabilities and numbers of features for use in modeling (e.g., the first six features were used in modeling, and *k* was 0.75 when generating the artificial Poisson point pattern). For each combination, after modeling with all possible subsets of the given features (except the empty set) and the parameters being estimated, an MML with six different Gaussian priors (zero means and variance equal to $1^{2},2^{2},5^{2},10^{2},20^{2},$ and $100^{2}$, respectively) were used to select the best models. Then, the same metrics, namely, the generalized Hamming distance defined by Equation (12), the Kullback–Leibler (KL) divergence, and the average log-likelihood of the prediction, were applied. This process was repeated 30 times for each combination.

Figure A5 shows that only in the case of four features given for modeling was there a noticeable difference in the average Hamming distance. In this case, features 1, 2, 3, and 4 were used for modeling. At the same time, the intensity function involved features 1, 3, 6, and 7, which means that features 1 and 3 were correlated and used for modeling, features 2 and 4 were not correlated and used for modeling, and features 6 and 7 were correlated but not used for modeling. We could observe that a larger variance in the priors led to a slightly lower generalized Hamming distance, which means that that the models selected by these metrics were closer to the true model. In addition to this case, when given the first six or eight features for modeling, there was no obvious difference among all these MML metrics.

Figure A6 and Figure A7 show that the difference was only significant given four features for modeling, and this was without considering how small the difference was compared to the actual log-likelihood/KL divergence values.

### Appendix A.2. Suitability of Bayesian Prior—Further Discussion

From the simulation results, we would like to argue that, even though $10^{2}$ is not the largest variance nor with the best or worst performance in the simulations, using such a variance in the priors does not give MML an advantage over using other variances. In the case of the four features given, there are obvious differences between the MML metrics using different variances in the prior, but $10^{2}$ is a variance with only moderate performance in all our measurements (generalized Hamming distance, average likelihood of prediction, and KL divergence). When six or eight features were used in modeling, the difference between using different variances was negligible. Thus, using $10^{2}$ as the variance of the priors for MML is a reasonable choice.
